# Numerical Solutions of Variable Coefficient Higher-Order Partial Differential Equations Arising in Beam Models

**DOI:** 10.3390/e24040567

**Published:** 2022-04-18

**Authors:** Abdul Ghafoor, Sirajul Haq, Manzoor Hussain, Thabet Abdeljawad, Manar A. Alqudah

**Affiliations:** 1 Institute of Numerical Sciences, Kohat University of Science and Technology, Kohat 26000, KP, Pakistan; abdulghafoor@kust.edu.pk; 2 Faculty of Engineering Sciences, GIK Institute, Topi 23640, KP, Pakistan; siraj@giki.edu.pk; 3 Department of Mathematics, Faculty of Sciences and Technology, Women University of Azad Jammu and Kashmir, Bagh 12500, AJK, Pakistan; manzoor366@gmail.com; 4 Department of Mathematics and Sciences, Prince Sultan University, Riyadh 11586, Saudi Arabia; 5 Department of Medical Research, China Medical University, Taichung 40402, Taiwan; 6 Department of Mathematical Sciences, Faculty of Sciences, Princess Nourah Bint Abdulrahman University, P.O. Box 84428, Riyadh 11671, Saudi Arabia; maalqudah@pnu.edu.sa

**Keywords:** higher-order PDEs, Euler–Bernoulli beam models, Haar wavelets, finite differences

## Abstract

In this work, an efficient and robust numerical scheme is proposed to solve the variable coefficients’ fourth-order partial differential equations (FOPDEs) that arise in Euler–Bernoulli beam models. When partial differential equations (PDEs) are of higher order and invoke variable coefficients, then the numerical solution is quite a tedious and challenging problem, which is our main concern in this paper. The current scheme is hybrid in nature in which the second-order finite difference is used for temporal discretization, while spatial derivatives and solutions are approximated via the Haar wavelet. Next, the integration and Haar matrices are used to convert partial differential equations (PDEs) to the system of linear equations, which can be handled easily. Besides this, we derive the theoretical result for stability via the Lax–Richtmyer criterion and verify it computationally. Moreover, we address the computational convergence rate, which is near order two. Several test problems are given to measure the accuracy of the suggested scheme. Computations validate that the present scheme works well for such problems. The calculated results are also compared with the earlier work and the exact solutions. The comparison shows that the outcomes are in good agreement with both the exact solutions and the available results in the literature.

## 1. Introduction

Small static and dynamic deflection problems can be observed properly in linear theory. For the determination of large (dynamic and static) deflection, linear theory is not beneficial and requires an accurate analysis. Linear theory admits inexact curvature in the study of beam deflections. The pioneering work in the field of thin beam theory was carried out by Bernoulli. Jacob Bernoulli then studied elastic theory and showed that the bending moment and curvature are both proportional. Later on, the Bernoulli theory was extended to loaded beams by Leonhard Euler.

The PDE of a thinand long beam is known as the Euler–Bernoulli model. The solution of this model shows the shortest distance (transverse vibration) from the beginning position in which stress and strain are linearly related. The mathematical form of the Euler–Bernoulli model [1] is described as follows:

(1) $$\gamma \left(x\right)\partial_{tt}u\left(x,t\right)+\partial_{xx}\left[C\left(x\right)\partial_{xx}u\left(x,t\right)\right]=E\left(x,t\right),\;0<x<1,\;0<t\leq T,$$

with appropriate initial and boundary conditions:

(2) $$u\left(x,0\right)=\Upsilon_{1}\left(x\right),\;\partial_{t}u\left(x,0\right)=\Upsilon_{2}\left(x\right),\;0\leq x\leq 1,$$

(3) $$\begin{array}{ccc} & & \left\{\begin{array}{c} u\left(0,t\right)=\varepsilon_{o}\left(t\right),\;\partial_{x}u\left(0,t\right)=\delta_{o}\left(t\right),\;0<t\leq T,\\ u\left(1,t\right)=\varepsilon_{1}\left(t\right),\;\partial_{x}u\left(1,t\right)=\delta_{1}\left(t\right),\;0<t\leq T, \end{array}\right. \end{array}$$

or

(4) $$\begin{array}{ccc} & & \left\{\begin{array}{c} u\left(0,t\right)=\zeta_{o}\left(t\right),\;\partial_{xx}u\left(0,t\right)=\mu_{o}\left(t\right),\;0<t\leq T\\ u\left(1,t\right)=\zeta_{1}\left(t\right),\;\partial_{xx}u\left(1,t\right)=\mu_{1}\left(t\right),\;0<t\leq T, \end{array}\right. \end{array}$$

where the notations $\partial_{tt}u\left(x,t\right),\;\partial_{xx}u\left(x,t\right)$ stand for the second derivative with respect to the time and space variables, respectively. Equation (1) is the variable coefficients’ FOPDE in which u(x,t) represents the beam displacement, γ(x) is the mass per unit length, C(x) shows the bending stiffness of the beam, E(x,t) is the source term, and *L* is the total length. This kind of equation has widespread applications in robotics designs and large flexible space structures [2,3]. Several analytical methods have been applied to derive the closed-form solution of the governing equation. Wazwaz [4] used the Adomian decomposition method to solve the variable coefficients’ FOPDEs. Lieu [5] applied He’s variational iteration technique to explain free vibration in an Euler–Bernoulli beam. For more generic cases of initial conditions, analytical solutions are quite complicated; therefore, researchers are constantly trying to focus on the numerical solutions. A variety of numerical methods via finite difference schemes have been developed for the solution of different forms of Equation (1) such as Jain et al. [6], Evans [7], Conte [8], Richtmyer [9], and Cranial [10]. Evans et al. [11] established a stable computational method using the hopscotch algorithm. Aziz et al. [12] proposed a three-level scheme using a parametric quintic spline to solve the FOPDEs. Rashidinia [13] implemented a three-level implicit scheme coupled with a sextic spline to solve fourth-order equations. Recently, Imtiaz et al. [14] numerically studied fractional-order Korteweg–de Vries and Burger’s equations via the meshless method. Senol and his co-authors [15] investigated the Coudrey–Dodd–Gibbon–Sawada–Kotera equation with three different methods. Akinyemi [16] solved (1+3)−dimensional fractional reaction–diffusion trimolecular models. The same author [17] and his collaborators computed the numerical solutions of coupled nonlinear Schrödinger–Korteweg-de Vries and Maccari systems numerically. Jiwari [18] applied barycentric rational interpolation and local radial basis function algorithms for the multi-dimensional Sine–Gordon equation.

In the past few years, wavelet-based approximation techniques gained great importance for solving PDEs [19,20]. In all kinds of wavelets, the simplest family is the Haar wavelet (HW), which comprises rectangular box functions. The usage of these wavelet attracted more researchers because of its easy implementation and achievements with good results. The HW based on Haar functions introduced by Alfred Haar in 1910, which are quite simple mathematically, are discontinuous at breaking points of the interval and, hence, not differentiable. Due to this reason, the direct implementation of the HW for the solution of differential equations is not possible. For this purpose, Cattani [21,22] used interpolating splines to remove this ambiguity. Similarly, an alternative idea was given by Chen and Hasio [23]. They suggested approximating the maximum-order derivative with finite HW series. Later on, this approach was extended for the solution of different problems. Lepik [24,25] introduced a numerical method using the HW to obtain the solution of PDEs. Jiwari [26] used the HW coupled with quasi-linearization for the solution of Burgers’ equation. Mittal et al. [27] studied numerically the system of viscous Burgers’ equations with the HW. Oruç [28,29] applied the finite difference hybrid scheme combined with the HW for the solution of modified Burgers’ and KdV equations. Kumar [30] solved the system of Burgers’ equations through the finite difference HW technique. Somayeh et al. [31,32] used a semi-analytical approach for solving the Hunter–Saxton and foam drainage equations. The same author [33] implemented an HW-based scheme for the solution of the two-dimensional system of PDEs. Mittal and Pandit [34] solved unsteady squeezing nano-fluid problems via the HW. Jiwari [35] developed a hybrid numerical method consisting of the HW for the nonlinear Burger’s equation. Pandit and Mitall [36] introduced the scale-3 Haar wavelet for the numerical solutions of the fractional advection dispersion equation. The same author and his co-authors [37] defined an operational-matrix-based algorithm for computational modeling of hyperbolic-type wave equations. For more details about these wavelet, we refer to [25,38].

In the present work, a hybrid scheme consisting of the HW and finite difference is suggested to find the numerical solution of Equation (1) with the homogeneous and non-homogeneous forms with variable coefficients. The remainder of this paper is as follows. In Section 2 the main motive of the current work is given. The preliminaries of the HW and their integrals are given in Section 3. The description of the method and stability are presented in Section 4 and Section 5, respectively. For the validation of the suggested scheme, some test problems are addressed in Section 6. In Section 7 we present the detail description of the initial disturbance and noisy data. Finally, the conclusion is reported in Section 8.

## 2. Motivation

To develop methods for solutions of higher-order PDEs either analytical, semi-analytical, or numerical is an essential need for the physical interpretation of the problem. In the literature, several numerical techniques have been adopted for FOPDEs. According to our knowledge, a hybrid numerical method based on the HW and finite difference along with stability for FOPDEs has not been reported yet. Therefore, the main motive of this work is to determine numerical solutions of FOPDEs using finite differences and the HW.

## 3. Haar Wavelets and Their Integrals

The HW based on Haar functions, which were defined in 1910, belongs to a well-known class of the wavelet family known as the Daubechies wavelet. The HW is a recent mathematical tool, which became popular in the numerical study of various differential and integral equations. Initially, it was introduced for the interval [0,1), but Lepik [24] extended this for any arbitrary interval [A,B).

To define the HW, assume x∈[A,B), then the HW family is defined for i≥1 as follows:

(5) $$\begin{array}{ccc} & & h_{1}\left(x\right)=\left\{\begin{array}{cc} 1, & x\in \left[A,B\right)\\ 0, & \mathrm{otherwise}. \end{array}\right. \end{array}$$

(6) $$\begin{array}{ccc} & & h_{i}\left(x\right)=\left\{\begin{array}{cc} 1, & x\in [\xi_{1}\left(i\right),\xi_{2}\left(i\right))\\ -1, & x\in [\xi_{2}\left(i\right),\xi_{3}\left(i\right)),\\ 0, & \mathrm{otherwise}, \end{array}\right. \end{array}$$

where $\xi_{s+1}\left(i\right)=A+\left(2k+s\right)\nu \delta x,\;s=0,1,2\;\mathrm{and}\;\nu =M/m$.

In the aforementioned equations, the interval is subdivided into 2M intervals of equal length $\delta x=\frac{B-A}{2M}$, where $M=2^{\lambda }$ and λ denote the maximal level of resolution. Next, two parameters j=0,⋯,λ and $k=0,\cdots ,2^{j}-1$ are taken, which decompose the wavelet number i=m+k+1, where $m=2^{j}$. To solve nth-order PDEs, we need the following repeated integrals:

(7) $$\begin{array}{c} R_{i,\beta }\left(x\right)=\int_{A}^{x}\int_{A}^{x}\cdots \int_{A}^{x}h_{i}\left(z\right)dz^{\beta }=\frac{1}{\left(\beta -1\right)!}\int_{A}^{x}{\left(x-z\right)}^{\beta -1}h_{i}\left(z\right)dz, \end{array}$$

where

β=1,2,⋯,n,i=1,2,⋯,2M.

Keeping in view Equations (5) and (6), the analytical expression of these integrals is given as [25]

(8) $$\begin{array}{ccc} & & R_{1,\beta }\left(x\right)=\frac{{\left(x-A\right)}^{\beta }}{\beta !}, \end{array}$$

(9) $$\begin{array}{ccc} & & R_{i,\beta }\left(x\right)=\left\{\begin{array}{cc} 0, & x<\xi_{1}\left(i\right)\\ \frac{1}{\beta !}{\left[x-\xi_{1}\left(i\right)\right]}^{\beta } & x\in \left[\xi_{1}\left(i\right),\xi_{2}\left(i\right)\right)\\ \frac{1}{\beta !}\left[{\left(x-\xi_{1}\left(i\right)\right)}^{\beta }-2({\left(x-\xi_{2}\left(i\right)\right)}^{\beta }\right] & x\in [\xi_{2}\left(i\right),\xi_{3}\left(i\right))\\ \frac{1}{\beta !}\left[{\left(x-\xi_{1}\left(i\right)\right)}^{\beta }-2({\left(x-\xi_{2}\left(i\right)\right)}^{\beta }+{\left(x-\xi_{3}\left(i\right)\right)}^{\beta }\right] & x\geq \xi_{3}\left(i\right). \end{array}\right. \end{array}$$

### Function Approximation

If $w\left(x\right)\in L^{2}\left[A,B\right)$ is a square integrable function, then it can be approximated via HW series as:

(10) $$w\left(x\right)=\sum_{i=1}^{2M}\alpha_{i}h_{i}\left(x\right),$$

where $\alpha_{i}$ are the unknown HW coefficients. At collocation points $x\to x_{l}$, Equation (10) takes the following discrete form:

(11) $$\tilde{w}=\sum_{i=1}^{2M}\alpha_{i}h_{i}\left(x_{l}\right).$$

In matrix form, Equation (11) can be written as:

(12) Θ=JA,

where Θ and A are 2M×1-dimensional matrices and J is a 2M×2M-dimensional matrix. From Equation (12), one can calculate the unknown coefficients, and then, the approximation to F(x) can be computed using Equation (10) for different resolution levels.

## 4. Description of the Method

In this section of the manuscript, the proposed scheme is presented for Equation (1) with boundary conditions in the form of Equation (3). We rewrite Equation (1) as:

(13) $$\gamma \left(x\right)\partial_{tt}u+C\left(x\right)\partial_{xxxx}u+2C^{\prime }\left(x\right)\partial_{xxx}u+C^{\prime \prime }\left(x\right)\partial_{xx}u=E,$$

where $C^{\prime }\left(x\right)=\frac{dC}{dx}$. Applying the finite difference to the temporal part and (θ-weighted ≤θ≤1 scheme, Equation (13) reduces to

(14) $$\begin{array}{c} \gamma \left(x\right)\frac{u^{𝚥+1}-2u^{𝚥}+u^{𝚥-1}}{\tau^{2}}+\theta {\left[C\left(x\right)\partial_{xxxx}u+2C^{\prime }\partial_{xxx}u+C^{\prime \prime }\left(x\right)\partial_{xx}u\right]}^{𝚥+1}\\ +\left(1-\theta \right){\left[C\left(x\right)\partial_{xxxx}u+2C^{\prime }\left(x\right)\partial_{xxx}u+C^{\prime \prime }\left(x\right)\partial_{xx}u\right]}^{𝚥}=E^{𝚥+1}, \end{array}$$

where $u^{𝚥}=u\left(x,t^{𝚥}\right),\;E^{𝚥}=E\left(x,t^{𝚥}\right),\;t^{𝚥+1}=\tau +t^{𝚥}$, and τ is the time step size. The associated boundary conditions Equation (3) is transformed to

(15) $$\begin{array}{ccc} & & \left\{\begin{array}{c} u^{𝚥+1}\left(0\right)=\varepsilon_{o}\left(t^{𝚥+1}\right),\;\partial_{x}u^{𝚥+1}\left(0\right)=\delta_{o}\left(t^{𝚥+1}\right),\;0<t\leq T\\ u^{𝚥+1}\left(1\right)=\varepsilon_{1}\left(t^{𝚥+1}\right),\;\partial_{x}u^{𝚥+1}\left(1\right)=\delta_{1}\left(t^{𝚥+1}\right),\;0<t\leq T. \end{array}\right. \end{array}$$

In more simplified form, Equation (14) can be written as

(16) $$\begin{array}{c} \gamma \left(x\right)u^{𝚥+1}+\tau^{2}\theta C\left(x\right)\partial_{xxxx}u^{𝚥+1}+2\theta \tau^{2}C^{\prime }\left(x\right)\partial_{xxx}u^{𝚥+1}+\theta \tau^{2}C^{\prime \prime }\left(x\right)\partial_{xx}u^{𝚥+1}=N^{𝚥}+\tau^{2}E^{𝚥+1}, \end{array}$$

where

$$\begin{array}{cc} N^{𝚥}= & \left(2u^{𝚥}-u^{𝚥-1}\right)\gamma \left(x\right)+\left(\theta -1\right)\tau^{2}{\left[C\left(x\right)\partial_{xxxx}u+2C^{\prime }\left(x\right)\partial_{xxx}u+C^{\prime \prime }\left(x\right)\partial_{xx}u\right]}^{𝚥}. \end{array}$$

Next, we assume the Haar wavelet approximation for the highest-order derivative as

(17) $$\begin{array}{cc} \partial_{xxxx}u^{𝚥+1}\left(x\right) & =\sum_{i=1}^{2M}\alpha_{i}^{𝚥+1}h_{i}\left(x\right), \end{array}$$

where $\alpha_{i}^{𝚥+1}$ are unknown constants to be determined. Integration of Equation (17) four times from 0 to *x* leads to:

(18) $$\begin{array}{cc} \partial_{xxx}u^{𝚥+1}\left(x\right) & =\sum_{i=1}^{2M}\alpha_{i}^{𝚥+1}R_{i,1}\left(x\right)+\partial_{xxx}u^{𝚥+1}\left(0\right)\\ \partial_{xx}u^{𝚥+1}\left(x\right) & =\sum_{i=1}^{2M}\alpha_{i}^{𝚥+1}R_{i,2}\left(x\right)+x\partial_{xxx}u^{𝚥+1}\left(0\right)+\partial_{xx}u^{𝚥+1}\left(0\right)\\ \partial_{x}u^{𝚥+1}\left(x\right) & =\sum_{i=1}^{2M}\alpha_{i}^{𝚥+1}R_{i,3}\left(x\right)+\frac{x^{2}}{2!}\partial_{xxx}u^{𝚥+1}\left(0\right)+x\partial_{xx}u^{𝚥+1}\left(0\right)+\partial_{x}u^{𝚥+1}\left(0\right),\\ u^{𝚥+1}\left(x\right) & =\sum_{i=1}^{2M}\alpha_{i}^{𝚥+1}R_{i,4}\left(x\right)+\frac{x^{3}}{3!}\partial_{xxx}u^{𝚥+1}\left(0\right)+\frac{x^{2}}{2!}\partial_{xx}u^{𝚥+1}\left(0\right)+x\partial_{x}u^{𝚥+1}\left(0\right)+u^{𝚥+1}\left(0\right). \end{array}$$

Using the boundary conditions $u^{𝚥+1}\left(1\right),\;\partial_{x}u^{𝚥+1}\left(1\right)$ in Equation (18), the unknown terms can be computed as

(19) $$\begin{array}{cc} \partial_{xxx}u^{𝚥+1}\left(0\right) & =\phi \left(t^{𝚥+1}\right)+12\sum_{i=1}^{2M}\alpha_{i}^{𝚥+1}R_{i,4}\left(1\right)-6\sum_{i=1}^{2M}\alpha_{i}^{𝚥+1}R_{i,3}\left(1\right)\\ \partial_{xx}u^{𝚥+1}\left(0\right) & =\psi \left(t^{𝚥+1}\right)-6\sum_{i=1}^{2M}\alpha_{i}^{𝚥+1}R_{i,4}\left(1\right)+2\sum_{i=1}^{2M}\alpha_{i}^{𝚥+1}R_{i,3}\left(1\right), \end{array}$$

where

$$\begin{array}{cc} \phi (t^{𝚥+1}) & =6\left[\delta_{1}\left(t^{𝚥+1}\right)+\delta_{o}\left(t^{𝚥+1}\right)-2\varepsilon_{1}\left(t^{𝚥+1}\right)+2\varepsilon_{o}\left(t^{𝚥+1}\right)\right]\\ \psi (t^{𝚥+1}) & =-2\left[\delta_{1}\left(t^{𝚥+1}\right)+2\delta_{o}\left(t^{𝚥+1}\right)\right]-6\left[\varepsilon_{o}\left(t^{𝚥+1}\right)-\varepsilon_{1}\left(t^{𝚥+1}\right)\right]. \end{array}$$

Making use of Equation (19) in Equation (18), we obtain

(20) $$\begin{array}{cc} \partial_{xxx}u^{𝚥+1}\left(x\right) & =\sum_{i=1}^{2M}\alpha_{i}^{𝚥+1}\left[R_{i,1}\left(x\right)-6R_{i,3}\left(1\right)+12R_{i,4}\left(1\right)\right]+\phi \left(t^{𝚥+1}\right)\\ \partial_{xx}u^{𝚥+1}\left(x\right) & =\sum_{i=1}^{2M}\alpha_{i}^{𝚥+1}\left[R_{i,2}\left(x\right)+\left(2-6x\right)R_{i,3}\left(1\right)+\left(12x-6\right)R_{i,4}\left(1\right)\right]+x\phi \left(t^{𝚥+1}\right)+\psi \left(t^{𝚥+1}\right)\\ \partial_{x}u^{𝚥+1}\left(x\right) & =\sum_{i=1}^{2M}\alpha_{i}^{𝚥+1}\left[R_{i,3}\left(x\right)+\left(2x-3x^{2}\right)R_{i,3}\left(1\right)+\left(6x^{2}-6x\right)R_{i,4}\left(1\right)\right]+\frac{x^{2}}{2}\phi \left(t^{𝚥+1}\right)\\ \; & +x\psi \left(t^{𝚥+1}\right)+\delta_{o}\left(t^{𝚥+1}\right)\\ u^{𝚥+1}\left(x\right) & =\sum_{i=1}^{2M}\alpha_{i}^{𝚥+1}\left[R_{i,4}\left(x\right)+\left(x^{2}-x^{3}\right)R_{i,3}\left(1\right)+\left(2x^{3}-3x^{2}\right)R_{i,4}\left(1\right)\right]+\frac{x^{3}}{3!}\phi \left(t^{𝚥+1}\right)\\ \; & +\frac{x^{2}}{2}\psi \left(t^{𝚥+1}\right)+x\delta_{o}\left(t^{𝚥+1}\right)+\varepsilon_{o}\left(t^{𝚥+1}\right). \end{array}$$

The proposed technique is based on the collocation approach; therefore, the collocation points are

$$x_{l}=\frac{l-0.5}{2M},\;l=1,\cdots ,2M.$$

Putting Equations (17) and (20) in Equation (16) and replacing *x* with $x_{l}$ leads to the following system of linear equations

(21) $$\begin{array}{c} \begin{array}{c} \sum_{i=1}^{2M}\alpha_{i}^{𝚥+1}[\gamma \left(x_{l}\right)A\left(i,l\right)+\tau^{2}\theta C\left(x_{l}\right)h_{i}\left(x_{l}\right)+2\theta \tau^{2}C^{\prime }\left(x_{l}\right)\Upsilon \left(i,l\right)+\tau^{2}\theta C^{\prime \prime }\left(x_{l}\right)\Psi \left(i,l\right)]\\ =\Lambda (i,𝚥+1)+\Omega (i,𝚥+1), \end{array} \end{array}$$

where

$$\begin{array}{cc} A(i,l)= & \left[R_{i,4}\left(x\right)+\left(x^{2}-x^{3}\right)R_{i,3}\left(1\right)+\left(2x^{3}-3x^{2}\right)R_{i,4}\left(1\right)\right]\\ \Upsilon (i,l)= & \left[R_{i,1}\left(x\right)-6R_{i,3}\left(1\right)+12R_{i,4}\left(1\right)\right]\\ \Psi (i,l)= & \left[R_{i,2}\left(x\right)+\left(2-6x\right)R_{i,3}\left(1\right)+\left(12x-6\right)R_{i,4}\left(1\right)\right]\\ \Lambda (l,t^{𝚥+1})= & N\left(l,t^{𝚥}\right)+E\left(l,t^{𝚥+1}\right)\\ \Omega (i,𝚥+1)= & -\gamma \left(x_{l}\right)\left\{\frac{x^{3}}{3!}\phi \left(t^{𝚥+1}\right)+\frac{x^{2}}{2}\psi \left(t^{𝚥+1}\right)+x\delta_{o}\left(t^{𝚥+1}\right)+\varepsilon_{o}\left(t^{𝚥+1}\right)\right\}\\ \; & -2\tau^{2}\theta C^{\prime }\left(x\right)\left\{\phi (t^{𝚥+1})\right\}-\tau^{2}\theta \left\{x\phi \left(t^{𝚥+1}\right)+\psi \left(t^{𝚥+1}\right)\right\}. \end{array}$$

There are 2M equations in Equation (21), which can be solved for 2M unknowns iteratively. After the computation of the unknown constants, the required solution can be computed from Equation (20).

### Note

To use the second kind of boundary conditions given in (Equation (4)), one may use first $u^{𝚥+1}\left(1\right),\;\partial_{xx}u^{𝚥+1}\left(1\right)$ in Equation (18) to obtain the following system of equations:

(22) $$\begin{array}{cc} \mu_{1}\left(t^{𝚥+1}\right) & =\sum_{i=1}^{2M}\alpha_{i}^{𝚥+1}R_{i,2}\left(1\right)+\partial_{xxx}u^{𝚥+1}\left(0\right)+\mu_{0}\left(t^{𝚥+1}\right)\\ \zeta_{1}\left(t^{𝚥+1}\right) & =\sum_{i=1}^{2M}\alpha_{i}^{𝚥+1}R_{i,4}\left(1\right)+\frac{1}{3!}\partial_{xxx}u^{𝚥+1}\left(0\right)+\frac{1}{2!}\mu_{0}\left(t^{𝚥+1}\right)+\partial_{x}u^{𝚥+1}\left(0\right)+\zeta_{0}\left(t^{𝚥+1}\right). \end{array}$$

After solving Equation (22), one can easily obtain $\partial_{xxx}u^{𝚥+1}\left(0\right)$$\partial_{x}u^{𝚥+1}\left(0\right)$. Once these terms are calculated, then expressions for the derivatives and solutions can be extracted following the strategies given in Equations (19)–(21).

## 5. Stability

Here, we present the theoretical result related to the stability of the proposed technique. To derive the condition, one can deduce the following equations from Equation (20) as:

(23) $$\partial_{xxxx}u^{𝚥+1}=H\alpha^{𝚥+1},\;\;\;\;\;\;\;\;\;\;\;$$

(24) $$\partial_{xxx}u^{𝚥+1}=\Upsilon \alpha^{𝚥+1}+{\tilde{\Upsilon }}^{𝚥+1},$$

(25) $$\;\partial_{xx}u^{𝚥+1}=\Psi \alpha^{𝚥+1}+{\tilde{\Psi }}^{𝚥+1},$$

(26) $$\;\;\;\;\;\;u^{𝚥+1}=F\alpha^{𝚥+1}+{\tilde{F}}^{𝚥+1},$$

where H,Υ,Ψ are differentiation matrices, F is the interpolation matrix, and ${\tilde{\Upsilon }}^{𝚥+1},{\tilde{\Psi }}^{𝚥+1},{\tilde{F}}^{𝚥+1}$ are boundary terms given in Equation (20). Using initial condition $\frac{u^{𝚥}-u^{𝚥-1}}{\tau }=\Upsilon_{2}\left(x\right)$ together with Equations (23)–(26) and $x\to x_{l}$ in Equation (16), we obtain

(27) $$\begin{array}{cc} \; & \left[\gamma \left(x_{l}\right)F\left(i,l\right)+\tau^{2}\theta C\left(x_{l}\right)H\left(i,l\right)+2\theta \tau^{2}C^{\prime }\left(x_{l}\right)\Upsilon \left(i,l\right)+\theta \tau^{2}C^{\prime \prime }\left(x_{l}\right)\Psi \left(i,l\right)\right]\alpha^{𝚥+1}=[\gamma \left(x_{l}\right)F\left(i,l\right)\\ & +\left(\theta -1\right)\tau^{2}\left(C\left(x_{l}\right)H\left(i,l\right)+2C^{\prime }\left(x_{l}\right)\Upsilon \left(i,l\right)+C^{\prime \prime }\left(x_{l}\right)\Psi \left(i,l\right)\right)]\alpha^{𝚥}+\tau^{2}E^{𝚥+1}+\tau \gamma \left(x_{l}\right)\Upsilon_{2}\left(x_{l}\right). \end{array}$$

In alternative form, Equation (27) can be written as:

(28) $$M\alpha^{𝚥+1}=N\alpha^{𝚥}+B^{𝚥+1},$$

where

$$M=\gamma \left(x_{l}\right)F\left(i,l\right)+\tau^{2}\theta C\left(x_{l}\right)H\left(i,l\right)+2\theta \tau^{2}C^{\prime }\left(x_{l}\right)\Upsilon \left(i,l\right)+\theta \tau^{2}C^{\prime \prime }\left(x_{l}\right)\Psi \left(i,l\right),$$

$$\;\;\;N=\gamma \left(x_{l}\right)F\left(i,l\right)+\left(\theta -1\right)\tau^{2}\left(C\left(x_{l}\right)H\left(i,l\right)+2C^{\prime }\left(x_{l}\right)\Upsilon \left(i,l\right)+C^{\prime \prime }\left(x_{l}\right)\Psi \left(i,l\right)\right),$$

$$B^{𝚥+1}=\tau^{2}E^{𝚥+1}+\tau \gamma \left(x_{l}\right)\Upsilon_{2}\left(x_{l}\right).\;\;\;\;\;\;\;\;\;\;\;\;\;\;\;\;\;\;\;\;\;\;\;\;\;\;\;\;\;\;\;\;\;\;\;\;\;\;\;\;\;\;\;\;\;\;\;\;\;\;\;\;\;\;$$

It follows from Equation (28) that

(29) $$\alpha^{𝚥+1}=M^{-1}N\alpha^{𝚥}+M^{-1}B^{𝚥+1}.$$

Plugging Equation (29) in Equation (26), one obtains

(30) $$\begin{array}{cc} u^{𝚥+1}= & F\left(M^{-1}N\alpha^{𝚥}+M^{-1}B^{𝚥+1}\right)+F^{𝚥+1}\\ = & FM^{-1}N\alpha^{𝚥}+FM^{-1}B^{𝚥+1}+F^{𝚥+1}. \end{array}$$

Using Equations (26) and (30), we have

(31) $$\begin{array}{cc} u^{𝚥+1} & =FM^{-1}N\left(F^{-1}u^{j}-F^{-1}{\tilde{F}}^{𝚥+1}\right)+FM^{-1}B^{𝚥+1}+F^{𝚥+1}\\ & =FM^{-1}NF^{-1}u^{j}-FM^{-1}NF^{-1}{\tilde{F}}^{𝚥+1}+FM^{-1}B^{𝚥+1}+F^{𝚥+1}. \end{array}$$

Equation (31) shows an iterative formula between $u^{𝚥+1}$ and $u^{𝚥}$. If ${\tilde{u}}^{𝚥}$ is the approximate solution, then

(32) $$\begin{array}{cc} {\tilde{u}}^{𝚥+1} & =FM^{-1}NF^{-1}{\tilde{u}}^{j}-FM^{-1}NF^{-1}{\tilde{F}}^{𝚥+1}+FM^{-1}B^{𝚥+1}+F^{𝚥+1}. \end{array}$$

Let $e^{𝚥}=\left|u^{𝚥}-{\tilde{u}}^{𝚥}\right|$, then from Equations (31) and (32),

(33) $$e^{𝚥+1}=\Xi e^{𝚥},$$

where $\Xi =FM^{-1}NF^{-1}$ is the amplification matrix. According to the Lax–Richtmyer criterion, the stability condition will be fulfilled if ||Ξ≤1||, which needs the spectral radius ρ(Ξ)≤1.

## 6. Illustrative Examples

In this section, we investigate the performance of the proposed method by solving various examples. To measure the efficiency, two error norms $L_{2},\;L_{\infty }$ were addressed, which are defined by:

$$\begin{array}{cc} L_{2} & ={\left(\sum_{i}^{2M}{\left|u_{i}^{ext}-{\tilde{u}}_{i}^{app}\right|}^{2}\right)}^{\frac{1}{2}}\\ L_{\infty } & =\max_{1\leq i\leq 2M}\left|u_{i}^{ext}-{\tilde{u}}_{i}^{app}\right|, \end{array}$$

where *u* and $\tilde{u}$ denote the exact and numerical solutions, respectively. Furthermore, we compute the convergence rate by using the following formula

$$\mathrm{Convergence}\;\mathrm{rate}=\frac{\log(e_{\infty }^{\lambda }-e_{\infty }^{\lambda +1})}{\log(2)}.$$

### 6.1. Problem 5.1

Consider Equation (1) with γ(x)=C(x)=1, in the following form

(34) $$\partial_{tt}u\left(x,t\right)+\partial_{xxxx}u\left(x,t\right)=\left(\pi^{4}-1\right)\sin\pi x\cos t,\;0\leq x\leq 1,\;\;0<t\leq T,$$

coupled with appropriate conditions

(35) $$u\left(x,0\right)=\sin\pi x,\;\partial_{t}u\left(x,0\right)=0,\;0\leq x\leq 1,$$

(36) $$u\left(0,t\right)=u\left(1,t\right)=0,\;\partial_{xx}u\left(0,t\right)=\partial_{xx}u\left(1,t\right)=0,\;\;0<t\leq T.$$

**Figure 1:**
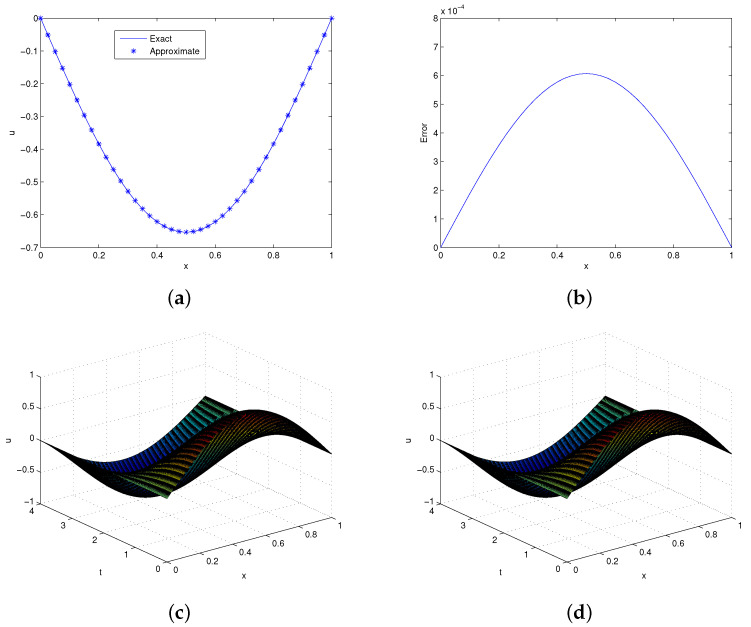
**Solutions profile of Problem 5.1**. (**a**) Exact and approximate solutions at t=4,τ=0.001. (**b**) Absolute error in (**a**). (**c**) Exact 3D plot. (**d**) Approximate 3D plot at t=4,τ=0.01,λ=4.

The exact solution of this problem is u(x,t)=sinπxcost. This problem was solved this problem with the help of the proposed method. In Table 1, pointwise errors are compared with existing results [12,39,40,41] for fixed τ=0.005 using different time and resolution levels. From the table, it is clear that the computed results are in good agreement with those available in the literature. Moreover, we calculated the convergence rate for this problem and addressed it in Table 2, which is approximately of order two. Graphical solutions in the form of 2D and 3D plots with the absolute error are shown in Figure 1 for time t=4. It is clear from the figure that the proposed method gives accurate solutions for a small number of collocation points and matches well the exact solution.

### 6.2. Problem 5.2

Consider the non-homogeneous problem of the form [41]

(37) $$\partial_{tt}u\left(x,t\right)+\partial_{xx}\left(\left(1+\sin\pi x\right)\partial_{xx}u\left(x,t\right)\right)=E\left(x,t\right),\;0\leq x\leq 1,\;\;0<t\leq T,$$

coupled with the initial conditions

(38) $$u\left(x,0\right)=\partial_{t}u\left(x,0\right)=0,\;0\leq x\leq 1,$$

and the boundary conditions

(39) $$u\left(0,t\right)=u\left(1,t\right)=0,\;\partial_{x}u\left(0,t\right)=\partial_{x}u\left(1,t\right)=0,\;\;0<t\leq T.$$

The corresponding source term can be adjusted according to the exact solution:

$$u\left(x,t\right)=x\left(1-x\right)\exp\left(-t\right)t^{2}\sin4\pi x.$$

**Figure 2:**
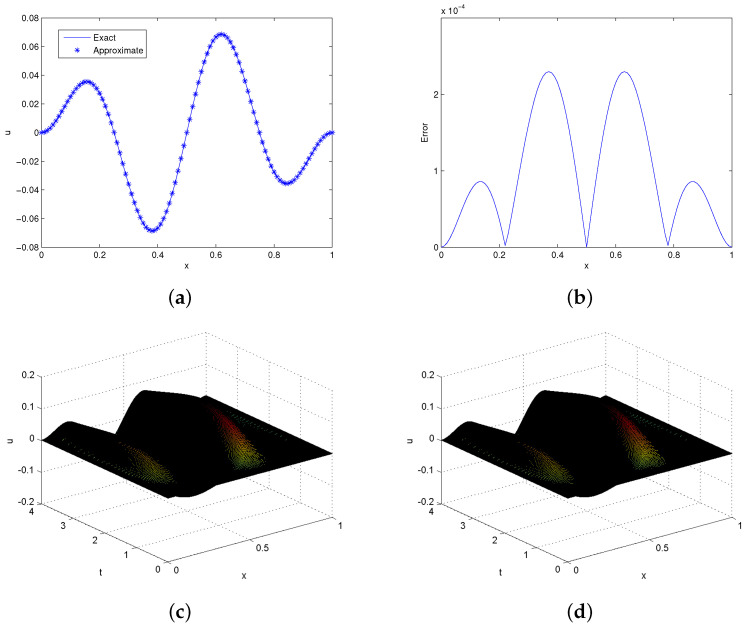
**Solutions profile of Problem 5.2**. (**a**) Exact and approximate solutions at t=4,τ=0.001. (**b**) Absolute error in (**a**). (**c**) Exact 3D plot. (**d**) Approximate 3D plot at t=4,τ=0.01,λ=5.

We obtained the solution of this problem in the time domain [0,4]. In Table 3, we record the absolute error at point x=0.5, using different times. From table, the computations show that our results are better than those described by Mohammadi [41]. In Table 4, different error norms are calculated, which identify that the proposed method produces good results at small resolution levels. The convergence rate of this problem was computed and is addressed in Table 5, which shows that the scheme is approximately of order two. Exact versus approximate solutions together with the absolute error are plotted at time t=4 in Figure 2. It is clear from the figure that the exact and approximate solutions are in good agreement.

### 6.3. Problem 5.3

Now, we consider the following equation:

(40) $$\partial_{tt}u\left(x,t\right)+\partial_{xx}\left(\left(1+\sin\pi x\right)\partial_{xx}u\left(x,t\right)\right)=E\left(x,t\right),\;0\leq x\leq 1,\;\;0<t\leq T,$$

inscribed with the initial conditions

(41) $$u\left(x,0\right)=\partial_{t}u\left(x,0\right)=0,\;0\leq x\leq 1,$$

and the boundary conditions

(42) $$\begin{array}{ccc} & & \left\{\begin{array}{c} u\left(0,t\right)=0,\;\partial_{x}u\left(0,t\right)=t^{2}e^{-t},\;0<t\leq T,\\ u\left(1,t\right)=t^{2}e^{-t},\;\partial_{x}u\left(1,t\right)=t^{2}e^{-t},\;0<t\leq T. \end{array}\right. \end{array}$$

The exact solution of this problem is given by

$$u\left(x,t\right)=\left(x+\sin^{3}\pi x\right)t^{2}e^{-t}.$$

**Figure 3:**
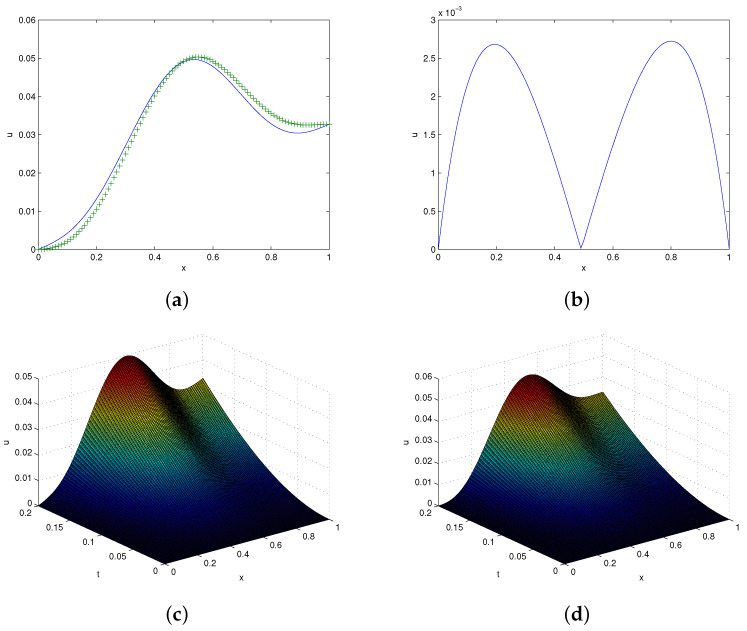
**Solutions profile of problem 5.3.** (**a**) Exact and approximate solutions at t=0.2,τ=0.001. (**b**) Absolute error in (**a**). (**c**) Exact 3D plot. (**d**) = Approximate 3D plot at t=0.2,τ=0.01,λ=5.

The approximate solution of this problem was computed at different resolution levels. In Table 6, we present the error norms for different times (t = 0.2, 0.5, 1, 4) using λ=4,5. It is observed from the table that the error norms are small, which shows the efficiency of the suggested scheme. The absolute error in displacement at x=0.5 matches those reported in [41] and presented in Table 7. It is clear from the table that the computed errors at different times are small. Moreover, the spectral radius for this problem and for previous two problems are addressed in Table 8 which shows that stability condition fulfilled. Graphical solutions and the absolute error are shown in Figure 3. From the figure, one can see that the exact and approximated solutions agree mutually.

## 7. Initial Disturbance and Noisy Data

Here, we discuss the effect of the small perturbation in the initial condition and the noisy data. In the initial perturbation, we take the initial condition $u_{0}=\Upsilon_{1}\left(x\right)+\epsilon$ where ϵ is a small number. The idea of this perturbation was taken from [42]. Simulations were performed for different values of resolution levels λ and and for different values of ϵ, which are given in Table 9. From the table, we concluded that the small perturbation in the initial data produces a small change in the solution, which shows that the method is stable. Similarly, we take $u_{0}\left(x_{i}\right)=\Upsilon_{1}\left(x_{i}\right)+{\left(-1\right)}^{\left(i\right)}\times \epsilon ,\;i=1,\cdots ,2M,$ for the noisy case [43]. In Table 10, we present the numerical results for the noisy case. From the table, it is pretty much clear that the variation in the resolution level for the noisy case still gives stable solutions.

**Figure 4:**
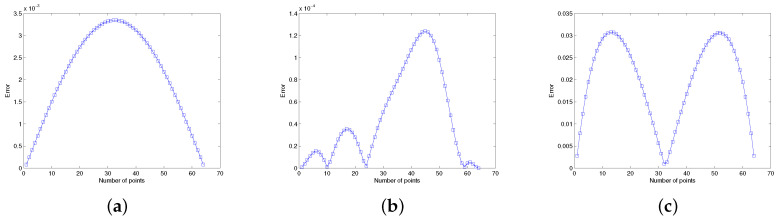
**Error profile of all problems at t=1.0**. (**a**) Error in Problem 5.1. (**b**) Error in Problem 5.2. (**c**) Error in Problem 5.3.

In both cases, when we increase the resolution level, the accuracy increases, which is obvious from Table 9 and Table 10. Besides this, the absolute error for the noisy case is plotted in Figure 4, which clearly indicates the accuracy of the techniques because this agrees with the previous error when no noise exists. The simulations and graphical error indicated that the method works for both types of data.

## 8. Conclusions

In this work, a mixed numerical method based on the Haar wavelet coupled with the finite difference was proposed to solve the FOPDEs. First, the scheme was tested with initial data, and then, small perturbations with and with out noise were introduced. The obtained results matched with earlier work and exact solutions. Furthermore, the accuracy of the scheme was checked via computing the $L_{2}$ and $L_{\infty }$ error norms. It was observed that the proposed scheme works well for smooth and noisy initial data, which indicates that the method can be applied for other such problems.

## Figures and Tables

**Table 1:** Absolute error at different points in Problem 5.1.

Methods	Points	*t*	x=0.1	x=0.2	x=0.3	x=0.4	x=0.5
Present	64	0.02	6.54 × 10${}^{-7}$	1.24 × 10${}^{-6}$	1.71 × 10${}^{-6}$	2.01 × 10${}^{-6}$	2.11 × 10${}^{-6}$
	64	0.05	5.22 × 10${}^{-6}$	9.94 × 10${}^{-6}$	1.36 × 10${}^{-5}$	1.60 × 10${}^{-5}$	1.69 × 10${}^{-5}$
	64	1	7.14 × 10${}^{-4}$	1.35 × 10${}^{-3}$	1.87 × 10${}^{-3}$	2.20 × 10${}^{-3}$	2.31 × 10${}^{-3}$
	128	0.02	2.60 × 10${}^{-7}$	4.94 × 10${}^{-7}$	6.81 × 10${}^{-7}$	8.00 × 10${}^{-7}$	8.42 × 10${}^{-7}$
	128	0.05	2.59 × 10${}^{-6}$	4.94 × 10${}^{-6}$	6.80 × 10${}^{-6}$	7.99 × 10${}^{-6}$	8.40 × 10${}^{-6}$
	128	1	6.83 × 10${}^{-4}$	1.29 × 10${}^{-3}$	1.78 × 10${}^{-3}$	2.10 × 10${}^{-3}$	2.21 × 10${}^{-3}$
Mittal [39]	91	0.02	3.20 × 10${}^{-5}$	6.08 × 10${}^{-5}$	8.37 × 10${}^{-5}$	9.84 × 10${}^{-5}$	1.04 × 10${}^{-4}$
	91	0.05	3.59 × 10${}^{-5}$	6.83 × 10${}^{-5}$	9.39 × 10${}^{-5}$	1.10 × 10${}^{-4}$	1.16 × 10${}^{-4}$
	91	1	6.32 × 10${}^{-5}$	1.20 × 10${}^{-4}$	1.65 × 10${}^{-4}$	1.94 × 10${}^{-4}$	2.04 × 10${}^{-4}$
	181	0.02	3.55 × 10${}^{-6}$	6.76 × 10${}^{-6}$	9.30 × 10${}^{-6}$	1.09 × 10${}^{-5}$	1.15 × 10${}^{-5}$
	181	0.05	3.99 × 10${}^{-6}$	7.58 × 10${}^{-6}$	1.04 × 10${}^{-5}$	1.23 × 10${}^{-5}$	1.29 × 10${}^{-5}$
	181	1	7.00 × 10${}^{-6}$	1.33 × 10${}^{-5}$	1.83 × 10${}^{-5}$	2.16 × 10${}^{-5}$	2.27 × 10${}^{-5}$
Caglar [40]	121	0.02	4.80 × 10${}^{-6}$	9.70 × 10${}^{-6}$	1.40 × 10${}^{-5}$	1.90 × 10${}^{-5}$	2.40 × 10${}^{-5}$
	191	0.02	5.20 × 10${}^{-6}$	2.10 × 10${}^{-6}$	3.10 × 10${}^{-6}$	4.20 × 10${}^{-6}$	5.20 × 10${}^{-6}$
	521	0.02	4.90 × 10${}^{-7}$	9.90 × 10${}^{-7}$	1.40 × 10${}^{-6}$	1.90 × 10${}^{-6}$	2.40 × 10${}^{-6}$
Aziz et al. [12]	20	0.05	9.30 × 10${}^{-6}$	8.00 × 10${}^{-6}$	2.80 × 10${}^{-6}$	1.00 × 10${}^{-6}$	2.70 × 10${}^{-6}$
Rashidinia [13]	20	0.05	2.91 × 10${}^{-6}$	1.73 × 10${}^{-6}$	1.60 × 10${}^{-6}$	2.23 × 10${}^{-6}$	2.60 × 10${}^{-7}$
Mohammadi [41]	40	0.05	2.96 × 10${}^{-6}$	1.77 × 10${}^{-6}$	1.64 × 10${}^{-6}$	2.28 × 10${}^{-6}$	2.65 × 10${}^{-7}$

**Table 2:** Convergence rate of Problem 5.1 at t=0.2.

Problem 4.1			
λ	τ	$L_{\infty }$	Rate
2	1/100	90,568 × 10${}^{-3}$	
3	1/200	2.4428 × 10${}^{-3}$	1.8904
4	1/400	6.8223 × 10${}^{-4}$	1.8402
5	1/800	2.0398 × 10${}^{-4}$	1.7418

**Table 3:** Absolute error in displacement of Problem 5.2.

Methods	Points	τ	t=0.2	t=0.4	t=0.8	t=1	t=2	t=4
Present	32	0.001	1.76 × 10${}^{-13}$	5.72 × 10${}^{-13}$	1.80 × 10${}^{-12}$	2.14 × 10${}^{-12}$	2.87 × 10${}^{-12}$	2.39 × 10${}^{-12}$
	64	0.001	1.72 × 10${}^{-13}$	5.75 × 10${}^{-13}$	1.48 × 10${}^{-12}$	1.90 × 10${}^{-12}$	2.95 × 10${}^{-12}$	6.13 × 10${}^{-13}$
[41]	100	0.01	1.78 × 10${}^{-5}$	5.85 × 10${}^{-5}$	1.57 × 10${}^{-4}$	2.00 × 10${}^{-4}$	2.95 × 10${}^{-4}$	1.60 × 10${}^{-4}$
	200	0.005	2.38 × 10${}^{-6}$	7.80 × 10${}^{-6}$	2.09 × 10${}^{-5}$	2.67 × 10${}^{-5}$	3.94 × 10${}^{-5}$	2.57 × 10${}^{-5}$

**Table 4:** Maximum error norms of Problem 5.2 at different times with τ=0.001.

	t=0.2		t=0.5		t=1		t=4	
λ	$L_{\infty }$	$L_{2}$	$L_{\infty }$	$L_{2}$	$L_{\infty }$	$L_{2}$	$L_{\infty }$	$L_{2}$
4	6.10 × 10${}^{-5}$	3.38 × 10${}^{-4}$	3.86 × 10${}^{-4}$	2.11 × 10${}^{-3}$	1.02 × 10${}^{-3}$	5.58 × 10${}^{-3}$	8.64 × 10${}^{-4}$	4.72 × 10${}^{-3}$
5	1.11 × 10${}^{-5}$	7.36 × 10${}^{-5}$	5.85 × 10${}^{-5}$	3.29 × 10${}^{-4}$	2.24 × 10${}^{-4}$	1.23 × 10${}^{-3}$	2.29 × 10${}^{-4}$	1.25 × 10${}^{-3}$

**Table 5:** Convergence rate of Problem 5.2 at t=1.

λ	τ	$L_{\infty }$	Rate
2	1/100	1.5254 × 10${}^{-2}$	
3	1/200	3.9824 × 10${}^{-3}$	1.9374
4	1/400	9.5864 × 10${}^{-4}$	2.0545
5	1/800	2.1397 × 10${}^{-4}$	2.1635

**Table 6:** Maximum error norms of Problem 5.3 at different times with τ=0.001.

	t=0.2		t=0.5		t=1		t=4	
λ	$L_{\infty }$	$L_{2}$	$L_{\infty }$	$L_{2}$	$L_{\infty }$	$L_{2}$	$L_{\infty }$	$L_{2}$
4	2.73 × 10${}^{-3}$	1.93 × 10${}^{-2}$	1.29 × 10${}^{-2}$	9.08 × 10${}^{-2}$	3.14 × 10${}^{-2}$	2.20 × 10${}^{-1}$	2.50 × 10${}^{-2}$	1.75 × 10${}^{-1}$
5	2.72 × 10${}^{-3}$	1.93 × 10${}^{-3}$	1.27 × 10${}^{-2}$	9.07 × 10${}^{-2}$	3.08 × 10${}^{-2}$	2.20 × 10${}^{-1}$	2.46 × 10${}^{-2}$	1.75 × 10${}^{-1}$

**Table 7:** Absolute error in displacement at x=0.5 of Problem 5.3.

Methods	Points	τ	t=0.2	t=0.4	t=0.8	t=1	t=2	t=4
Present	32	0.01	1.40 × 10${}^{-3}$	1.51 × 10${}^{-3}$	1.46 × 10${}^{-4}$	6.95 × 10${}^{-4}$	3.62 × 10${}^{-3}$	2.68 × 10${}^{-3}$
[41]	100	0.01	7.82 × 10${}^{-3}$	2.59 × 10${}^{-2}$	7.27 × 10${}^{-2}$	9.43 × 10${}^{-2}$	1.44 × 10${}^{-1}$	7.65 × 10${}^{-2}$

**Table 8:** Spectral radius of Problems 5.1, 5.2, and 5.3 at t=0.2.

λ	Problem 5.1	Problem 5.2	Problem 5.3
	ρ(Ξ)	ρ(Ξ)	ρ(Ξ)
1	0.99984	0.99937	0.99937
2	0.99984	0.99929	0.99929
3	0.99984	0.99927	0.99927
4	0.99984	0.99927	0.99927

**Table 9:** Maximum error norms of all problems for t=1.0, after initial disturbances.

$\epsilon =10^{-2}$	Problem 5.1		Problem 5.2		Problem 5.3	
λ	$L_{\infty }$	$L_{2}$	$L_{\infty }$	$L_{2}$	$L_{\infty }$	$L_{2}$
1	4.53 × 10${}^{-2}$	2.02 × 10${}^{-1}$	4.82 × 10${}^{-1}$	3.16 × 10${}^{0}$	1.30 × 10${}^{-1}$	2.27 × 10${}^{-1}$
2	2.10 × 10${}^{-2}$	9.41 × 10${}^{-2}$	1.67 × 10${}^{-2}$	8.62 × 10${}^{-2}$	4.90 × 10${}^{-2}$	3.09 × 10${}^{-1}$
3	1.46 × 10${}^{-2}$	6.55 × 10${}^{-2}$	4.14 × 10${}^{-3}$	2.18 × 10${}^{-2}$	3.40 × 10${}^{-2}$	2.26 × 10${}^{-1}$
4	1.30 × 10${}^{-2}$	5.82 × 10${}^{-2}$	1.34 × 10${}^{-3}$	5.94 × 10${}^{-3}$	3.15 × 10${}^{-2}$	2.20 × 10${}^{-1}$
$\epsilon =10^{-3}$	Problem 5.1		Problem 5.2		Problem 5.3	
λ	$L_{\infty }$	$L_{2}$	$L_{\infty }$	$L_{2}$	$L_{\infty }$	$L_{2}$
1	3.64 × 10${}^{-2}$	1.63 × 10${}^{-1}$	4.78 × 10${}^{-1}$	3.16 × 10${}^{0}$	1.36 × 10${}^{-1}$	7.63 × 10${}^{-1}$
2	1.18 × 10${}^{-2}$	5.30 × 10${}^{-2}$	1.55 × 10${}^{-2}$	8.56 × 10${}^{-2}$	5.02 × 10${}^{-2}$	3.16 × 10${}^{-1}$
3	5.38 × 10${}^{-3}$	2.40 × 10${}^{-2}$	3.99 × 10${}^{-3}$	2.18 × 10${}^{-2}$	3.39 × 10${}^{-2}$	2.26 × 10${}^{-1}$
4	3.75 × 10${}^{-3}$	1.67 × 10${}^{-2}$	9.02 × 10${}^{-4}$	4.70 × 10${}^{-3}$	3.13 × 10${}^{-2}$	2.20 × 10${}^{-1}$

**Table 10:** Maximum error norms of all problems for $t=1.0\;\epsilon =10^{-2},$ with noisy initial data.

Noise = 1%	Problem 5.1		Problem 5.2		Problem 5.3	
λ	$L_{\infty }$	$L_{2}$	$L_{\infty }$	$L_{2}$	$L_{\infty }$	$L_{2}$
1	3.55 × 10${}^{-2}$	1.58 × 10${}^{-1}$	4.77 × 10${}^{-1}$	3.16 × 10${}^{0}$	1.37 × 10${}^{-1}$	7.67 × 10${}^{-1}$
2	1.08 × 10${}^{-2}$	4.84 × 10${}^{-2}$	1.54 × 10${}^{-2}$	8.56 × 10${}^{-2}$	5.03 × 10${}^{-2}$	3.16 × 10${}^{-1}$
3	4.35 × 10${}^{-3}$	1.94 × 10${}^{-2}$	3.98 × 10${}^{-3}$	2.18 × 10${}^{-2}$	3.39 × 10${}^{-2}$	2.26 × 10${}^{-1}$
4	2.72 × 10${}^{-3}$	1.21 × 10${}^{-2}$	8.47 × 10${}^{-4}$	4.69 × 10${}^{-3}$	3.13 × 10${}^{-2}$	2.20 × 10${}^{-1}$

## Data Availability

If interested readers need the data, please contact the first author via email.

## References

[1] Ahn J., Stewart D.E. (2007). An Euler–Bernoulli beam with dynamic frictionless contact: Penalty approximation and existence. Numer. Funct. Anal. Optim..

[2] Kunisch K., Graif E. (1985). Parameter estimation for the Euler–Bernoulli beam. Mat. Apficada Comput..

[3] Timoshenko S.P., Gere J.M. (1961). Theory of Elastic Stability.

[4] Wazwaz A.M. (2001). Analytic treatment for variable coefficient fourth-order parabolic partial differential equations. Appl. Math. Comput..

[5] Liu Y., Gurram C.S. (2009). The use of Hes variational iteration method for obtaining the free vibration of an EulerBernoulli beam. Math. Comput. Model..

[6] Jain M.K., Iyengar S.R.K., Lone A.G. (1976). Higher order difference formulas for a fourth order parabolic partial differential equation. Int. J. Numer. Methods Eng..

[7] Evans D.J. (1965). A stable explicit method for the finite difference solution of a fourth order parabolic partial differential equation. Comput. J..

[8] Conte S.D. (1957). A stable implicit finite difference approximation to a fourth order parabolic equation. J. Assoc. Comput. Mech..

[9] Richtmyer R.D., Morton K.W. (1967). Difference Methods for Initial Value Problems.

[10] Crandall S.H. (1954). Numerical treatment of a fourth order partial differential equations. J. Assoc. Comput. Mech..

[11] Danaee A., Khan A., Khan I., Aziz T., Evans D.J. (1982). Hopscotch procedure for a fourthorder parabolic partial differential equation. Math. Comput. Simul..

[12] Aziz T., Khan A., Rashidinia J. (2005). Spline methods for the solution of fourth-order parabolic partial differential equations. Appl. Math. Comput..

[13] Rashidinia J., Mohammadi R. (2010). Sextic spline solution of variable coefficient fourthorder parabolic equations. Int. J. Comput. Math..

[14] Ahmad I., Hijaz A., Inc M., Rezazadeh H., Akbar M.A., Khater M.M.A., Akinyemi L., Jhangeer A. (2021). Solution of fractional-order Korteweg-de Vries and Burgers’ equations utilizing local meshless method. J. Ocean Eng. Sci..

[15] Senol M., Akinyemi L., Ata A., Iyiola O.S. (2021). SApproximate and generalized solutions of conformable type Coudrey–Dodd–Gibbon–Sawada–Kotera equation. Int. J. Mod. Phy. B.

[16] Akinyemi L., Iyiola O.S. (2021). Analytical Study of (3+1)-Dimensional Fractional-Reaction Diffusion Trimolecular Models. Int. J. Appl. Comp. Math..

[17] Akinyemi L., Veeresha P., Ajibola S.O. (2021). Numerical simulation for coupled nonlinear Schrödinger–Korteweg–de Vries and Maccari systems of equations. Mod. Phy. Lett. B.

[18] Jiwari R. (2021). Barycentric rational interpolation and local radial basis functions based numerical algorithms for multidimensional sine-Gordon equation. Num. Meth. Part. Diff. Eqs..

[19] Bertoluzza S., Dahmen W., Kurdila A.J., Oswald P. (1977). An adaptive collocation method based on interpolating wavelets. Multi-Scale Wavelet Methods for Partial Differential Equations.

[20] Beylkin G., Keiser J.M., Dahmen W., Kurdila A.J., Oswald P. (1977). An adaptive pseudo-wavelet approach for solving nonlinear partial differential equations. Multi-Scale Wavelet Methods for Partial Differential Equations.

[21] Cattani C. (2001). Haar wavelet splines. J. Interdiscip. Math..

[22] Cattani C. (2004). Haar wavelets based technique in evolution problems. Proc. Estonian Acad. Sci. Phys. Math..

[23] Chen C.F., Hasio C.H. (1997). Haar wavelet method for solving lumped and distributedparameter systems. IEE Proc. Number Control Theory Appl..

[24] Lepik U. (2007). Numerical solution of evolution equations by the Haar wavelet method. Appl. Math. Comput..

[25] Lepik U. (2011). Solving PDEs with the aid of two-dimensional Haar wavelets. Comput. Math. Appl..

[26] Jiwari R. (2012). A Haar wavelet quasilinearization approach for numerical simulation of Burgers equation. Comput. Phy. Comm..

[27] Mittal R.C., Kaur H., Mishra V. (2015). Haar Wavelet Based Numerical Investigation of Coupled Viscous Burgers equation. Int. J. Comput. Appl..

[28] Oruç Ö., Bulut F., Esen A. (2015). A Haar wavelet-finite difference hybrid method for the numerical solution of the modified Burgers equation. J. Math. Chem..

[29] Oruç Ö., Bulut F., Esen A. (2016). Numerical solution of the KdV equation by Haar wavelet method. Pramana J. Phys..

[30] Kumar M., Pandit S. (2014). A composite numerical scheme for the numerical simulation of coupled Burgers equation. Comput. Phys. Commun..

[31] Arbabi S., Nazari A., Darvishi M.T. (2016). A semi-analytical solution of Hunter-Saxton equation. Optik.

[32] Arbabi S., Nazari A., Darvishi M.T. (2016). A semi-analytical solution of foam drainage equation by Haar wavelets method. Optik.

[33] Arbabi S., Nazari A., Darvishi M.T. (2017). A two-dimensional Haar wavelets method for solving systems of PDEs. Appl. Math. Comp..

[34] Mittal R.C., Pandit S. (2017). Numerical simulation of unsteady squeezing nanofluid and heat flow between two parallel plates using wavelets. Int. J. Ther. Sci..

[35] Jiwari R. (2015). A hybrid numerical scheme for the numerical solution of the Burgers’ equation. Comput. Phys. Commun..

[36] Pandit S., Mittal R.C. (2020). A numerical algorithm based on scale-3 Haar wavelets for fractional advection dispersion equation. Eng. Comput..

[37] Pandit S., Jiwari R., Bedi K., Koksal M.E. (2017). Haar wavelets operational matrix based algorithm for computational modeling of hyperbolic type wave equations. Eng. Comput..

[38] Haq S., Ghafoor A. (2018). An efficient numerical algorithm for multi-dimensional time dependent partial differential equations. Comput. Math. Appl..

[39] Mittal R.C., Jain R.K. (2011). B-Splines methods with redefined basis functions for solving fourth order parabolic partial differential equations. Appl. Math. Comput..

[40] Caglar H., Caglar N. (2008). Fifth-degree B-spline solution for a fourth-order parabolic partial differential equations. Appl. Math. Comput..

[41] Mohammadi R. (2014). Sextic B-spline collocation method for solving Euler–Bernoulli Beam Models. Appl. Math. Comput..

[42] Shivanian E., Jafarabadi A. (2017). Inverse Cauchy problem of annulus domains in the framework of spectral meshless radial point interpolation. Eng. Comput..

[43] Solodusha S.V., Mokry I.V. (2016). A numerical solution of one class of Volterra integral equations of the first kind in terms of the machine arithmetic features. Bull. SUSU MMCS.

